# Predicting the quality of life based on pain dimensions and psychiatric symptoms in patients with Painful diabetic neuropathy: a cross-sectional prevalence study in Iranian patients

**DOI:** 10.1186/s12955-021-01697-w

**Published:** 2021-02-09

**Authors:** Mohammadreza Davoudi, Parnian Rezaei, Fereshteh Rajaeiramsheh, Seyed Majid Ahmadi, Amir Abbas Taheri

**Affiliations:** 1 Department of Clinical Psychology, Faculty of Behavioral Science, University of Social Welfare and Rehabilitation Sciences, Tehran, Iran; 2 Department of Clinical Psychology, Shahid Beheshti University, Tehran, Iran; 3 Department of Internal Medicine, School of Medical, Yasouj University of Medical Sciences (YUMS), Yasouj, Iran; 4 Department of Counseling, Faculty of Behavioral Science, University of Social Welfare and Rehabilitation Sciences, Tehran, Iran

**Keywords:** Diabetes mellitus, Anxiety, Depression, Pain, Diabetic neuropathy, Quality of life, Sleep hygiene

## Abstract

**Background:**

This study aimed to predict the quality of life (QOL) in patients with Painful Diabetic Neuropathy (PDN) based on pain severity, pain catastrophizing, pain acceptance, depression, anxiety, and sleep disturbance. Also, this study was aimed to assess the prevalence of psychiatric symptoms in Iranian patients with PDN.

**Method:**

1120 patients (mean age, 53.6 ± 12.6 years) participated in the research. Data were collected by the Quality of life questionnaire (NeuroQoL); Beck Depression Inventory, Beck Anxiety Inventory, the visual analog scale for pain severity, Pain Catastrophizing Scale (PCS), Chronic Pain Acceptance Questionnaire (CPAQ) and Pittsburgh Sleep Quality Index (PSQI). Finally, the data were analyzed using SPSS-26 by multiple regression analysis.

**Results:**

The results showed the regression models’ significance, and the dependent variables predicted 42% of total changes in the QOL. The most significant predicting factors were depression, pain catastrophizing, pain acceptance, pain severity, sleep disturbance, and anxiety in order. In patients with PDN, the prevalence of sleep disturbances, depression, and anxiety were 85.5%, 68.2%, and 62.1%, respectively. Also, comorbid depression and anxiety were found in 47% of patients.

**Conclusion:**

Results demonstrated a significant relationship between pain-related and psychiatric dimensions with QOL. Thus, it is suggested to design more specific psychological-based rehabilitation interventions in which these variables are considered. They should focus on more significant variables (such as depression and pain catastrophizing) to reach better treatment outcomes. Furthermore, this research shows a high level of anxiety, depression, and sleep disturbance in Iranian patients with PDN. Thus, experts and clinicians are suggested to focus on reducing these psychiatric symptoms.

## Introduction

Diabetes is known as the silent killer [1], which occurs when the pancreas can no longer make insulin or the body cells don't appropriately respond to the produced insulin [2]. The International Diabetes Federation (IDF) reported that more than 436 million people have diabetes all around the world [2, 3]. Diabetes is associated with a wide range of health issues, including fatigue, irritability, recurrent infections, ketoacidosis, brain ischemia, nephropathy, and mental health disorders [4, 5]. Painful diabetic neuropathy (PDN) is among the most common complications in a patient with chronic Diabetes Mellitus (type 1 and 2). PDN manifests with an intense pain arising as a direct consequence of the disease or lesions affecting the peripheral nervous system (PNS) [6]. Above 30% of patients with diabetes diagnosed with PDN. These patients report a stinging, burning, and keen sensation that increases at night with a loss of sensation or numbness of the involved area [6, 7]. Painful diabetic neuropathy is linked with mood instability, interpersonal problems, psychiatric symptoms, general activity deficits, life disenjoyment, and a generally reduced quality of life [8, 9]. Moreover, patients with PDN experience severe pain, which is debilitating and leads to dissatisfaction, fatigue, and distress, ultimately reducing the QoL. This pain occurs in varying degrees and intensifies at night. Due to its debilitating properties, neuropathic pain causes dissatisfaction, fatigue, and distress; ultimately reduces the QoL of these patients much more than other patients with diabetic [6, 9]. Besides pain severity, pain interpretation plays a significant role in the QoL of patients. Pain Catastrophizing is the mental sensation of losing physical activity due to pain severity. In pain catastrophizing, the level of pain-related disability is estimated much higher than the actual level, which is associated with increased disability and decreased QOL [10]. Pharmacological and psychological interventions, at best, relieve only 50% of pain severity [11].Thus, clinicians should consider to pain acceptance and pain catastrophizing for increasing QoL [12, 13]. The primary goal of early diabetes diagnosis and treatment is improving the QoL of patients; thus, numerous studies have assessed the effectiveness of different therapeutic methods to improve the QoL of patients with PDN [13, 14]. Previous studies show the higher prevalence of psychological disorders, most commonly depression (15–50%) and anxiety (8–60%), in patients with PDN, which reduce QoL, life expectancy, and treatment effectiveness. Also, more than 35% of patients with PDN experience comorbid depression and anxiety [14–16]. Many studies suggest that these symptoms significantly reduce QoL [16, 17]. Sleep-related issues are also common in these patients, with a prevalence of more than 40%. These problems are highly associated with reduced QoL. Moreover, the lack of sleep disorder improvement in these patients is an obstacle to improving other symptoms [16].

Conclusively, problems related to pain, depression, anxiety, and sleep disturbance severely reduce the QoL (as a primary goal of therapeutic interventions) in patients with PDN. These symptoms have been investigated in several studies [6, 9, 12, 18], though these studies have various limitations, including small sample size (less than 60 participants) [19, 20], lack of individual interviews [10], and lack of psychological variables examination [21].

On the other hand, the prevalence of diabetes is more than 15% in Iran's general population [22]. The prevalence of these psychiatric issues has not to be investigated in Iran in diabetic patients with PDN with suitable sample size and a structured interview. Thus, this study aimed to estimate the QoL in patients with PDN based on pain severity, pain catastrophizing, pain acceptance, depression, anxiety, and sleep disturbance. Besides, the prevalence of psychiatric problems is assessed in Iranian patients with PDN.

## Methods

### Study design and research criteria

We conducted a cross-sectional study of patients with painful diabetic neuropathy in Tehran, the Capital of Iran, from November 1, 2019, to August 1, 2020.

Inclusion criteria include (1) Willingness to participate in research, (2) ranging in age between 18 and 70, (3) Diagnosed with PDN by a neurologist, (4) No history of hospitalization in psychiatric wards, and (5) No history or current diagnosis of substance abuse. Exclusion criteria include (1) dissatisfaction with entering the research, and (2) Failure to complete the questionnaires.

### Procedure

Based on the convenience sampling method, 1500 patients with PDN were selected to participate in this research. Each patient had medical records in one of the specialized diabetes treatment centers in Tehran Province. All of them had received PDN diagnosis and were under treatment. They were contacted and explained about the study. Extensive explanations were given about the study. Then each individual was given an appointment at the clinic to complete the tools of the research. Anonymous paper surveys were utilized for gathering data. After screening based on criteria, an appointment was made for each patient. These evaluations were held by five psychologists separately (to speed up the assessment process). Patients separately answered the questionnaire booklet under the five psychologists' administration and supervision (blinded about research aims). Besides, written informed consent was taken from all patients.

**Figure 1:**
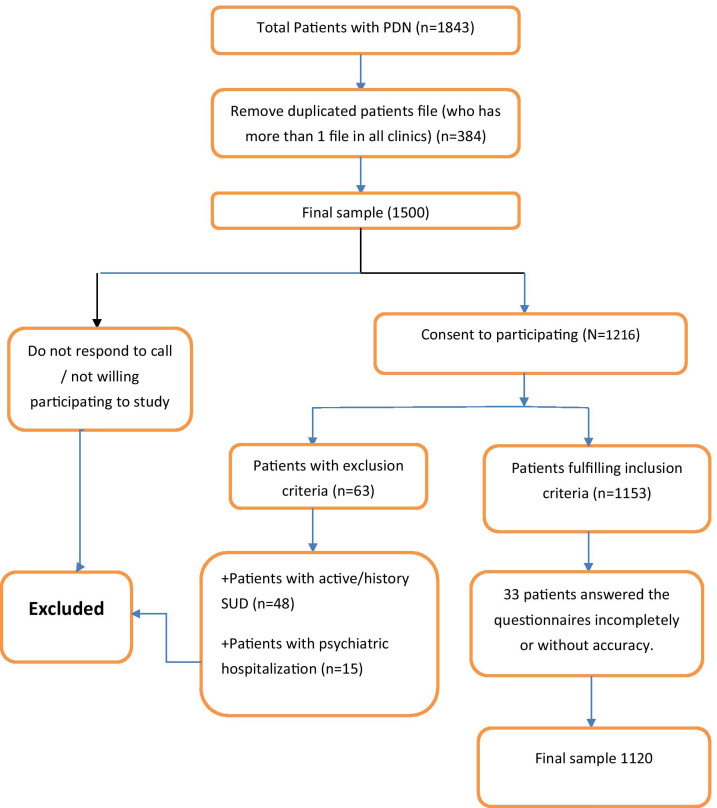
Flowchart diagram of study

Of these 1500 patients, 284 neither went to the center nor responded to the researchers' calls; Forty-eight patients had a history of substance use or were taking it; Fifteen had a history of hospitalization in a psychiatric hospital, reported in psychiatric hospitals or had traumatic brain injury; Thirty-three people answered the questionnaires incompletely or without accuracy. Finally, 1120 people entered the results analysis process. Figure 1 presented flowchart diagram of the study selection.

### Assessments

#### Quality of life

The Neuropathy Specific Quality of life questionnaire (NeuroQoL) is an appropriate validated measure for neuropathic QoL. It has 27 items. NeuroQoL assesses diabetic neuropathy-related emotional and physical problems affecting diurnal life and well-being. NeuroQoL consists six subscales: painful symptoms, dependence on others, emotional distress, unsteadiness while walking or standing, restriction in daily activities, paresthesia, interpersonal problems, and decreased or lack of ability to feel the temperature. NeuroQoL reliably captures the critical aspects of the patients’ experience of PDN and is a valid instrument for examining the impact of neuropathy on QOL [23]. The reliability of the subscales ranged from 0.86 to 0.95. As four items repeated in more than one subscale, the total score is 31–155. Persian Version of NeuroQoL has demonstrated high psychometric properties with Cronbach Alpha 0.87 assessed by authors of the current paper.

#### Pain severity

The severity of pain of the subjects was assessed by a 10-cm visual analog scale (VAS) where the "0" means not feeling any pain, and "10" shows unbearable severe pain [24].

#### Pain catastrophizing

Pain Catastrophizing Scale (PCS) was used to assess pain catastrophizing in PDN patients. The PCS is a 13-item self-report scale developed to assess individuals’ rate of catastrophic beliefs related to painful experiences. PCS ranged on a 5-point Likert scale (0 = not at all to 4 = always). Higher scores mean greater pain catastrophizing. The PCS has shown good psychometric properties for clinical /non-clinical Iranian samples (Cronbach’s alpha = 0.88) [25, 26].

#### Pain acceptance

Chronic Pain Acceptance Questionnaire (CPAQ) was used to assess pain acceptance. The CPAQ is a 20 item assessment tool for chronic pain that each subject should answer every item in a 7-point Likert scale. Each item scored on a 7-point Likert scale, ranging from 0 (never) to 6 (always), and the items for the pain satisfaction scale scored reversely, ranging from 0 to 120. Higher scores indicate higher levels of pain acceptance. The Persian version of the CPAQ also has shown adequate psychometric properties. Assessment of psychometric properties of the Persian version has shown that the value of coefficient was 0.89 and the value of test–retest reliability was 0.71[12].

#### Depression

Second edition of the Beck Depression Inventory (BDI-II) is a 21-item scale for assessing depression. Each item scored on a 4-point Likert scale, ranging from 0 (never) to 3 (always). The minimum score in this test can be 0, and the maximum can be 63. The cut-off scores of BDI include minimal or nothing depression (0–13), mild depression (14–19), moderate depression (20–28), and severe (above 29). In the BDI.II, the cut-off point for adulthood depression in the medical context is 14 [27].BDI is utilized for measuring depressed patients’ signs and symptoms. The test–retest correlation coefficient of this scale was 0.93. BDI has been widely used in various countries and demonstrated a suitable application Its Persian version also has shown acceptable validity and reliability [28, 29].

#### Anxiety

Beck Anxiety Inventory (BAI) is also used for assessing anxiety. BAI is a 21-item scale that is scored on a Likert scale from zero to three. Each item represents one of the most prevalent symptoms of anxiety; the total score ranges from 0 to 63. The Cronbach’s alpha of the English version of BAI has been reported to be 0.82. Researches have demonstrated that the Persian version of BAI has high-grade reliability (r = 0.72), a suitable validity (r = 0.83), and an excellent internal consistency (a = 0.92) [30]. According to the manual, the suggested cutoff for clinically significant anxiety is 16. The BAI category includes no anxiety (0–16), moderate anxiety (17–35), and severe anxiety (36–63) [31, 32].

#### Sleep disturbance

Pittsburgh Sleep Quality Index (PSQI) is used for assessing sleep quality. PSQI contains 18 items related to sleep quality in the past month. The total score ranges from zero (without any sleep disturbance) to 21 (wholly impaired sleep). The value of reliability is 0.83. PSQI has an average sensitivity of 90% and specificity of more than 86% for identifying cases with a sleep disorder, using a cut-off score of five. In the Iranian psychiatrically healthy community, the value of Cronbach’s alpha coefficient is 0.78, and the sensitivity coefficient is more than 0.95.[9, 33].

### Statically analysis

Pearson’s correlation and multiple linear regression models were employed to investigate the relationship between variables. SPSS version 26 statistical package for social sciences was utilized for analyzing data. The Kolmogorov–Smirnov test was applied for normalizing data. Homoscedasticity was examined using the scatter plot. Multiple outliers were evaluated by Mahalanobis distance.

## Results

### Essential characteristics of the study population

Five hundred sixty patients with PDN participated in this study. The mean age of the subjects was 53.6 ± 12.6 years, of which more than half were male (342 patients, 61.1% of them) and married (90.9%). Also, the mean duration of their diabetes was 13.3 ± 3.4 (Table 1).

**Table 1:** Demographic information of participants

Variables	Status	Free count (%)
Gender	Male	684 (61.1)
	Female	436 (38.9)
Marital status	Married	966 (86.3)
	Single	18 (1.6)
	Other statuses	136 (12.1)
Education level	Under diploma	778 (69.5)
	Diploma	316 (28.2)
	University	26 (2.3)
Insulin treatment	Yes	798 (71.3)
	No	322 (28.7)

All of the patients were residents of Isfahan, Iran, and were Persian‑native speakers. The mean and standard deviation of the variables are reported in Table 2. Independent t‑test showed that no significant difference between the mean scores of QoL, pain severity, pain acceptance, depression, pain catastrophizing, and sleep disturbance in two groups of males and females (P > 0.05).

**Table 2:** Descriptive statics of research variables

Variable		Mean ± SD		P value
	Total	Male	Female	
Depression	18.61 ± 8.5	18.1 ± 8.5	19.3 ± 8.4	0.1
Anxiety	20.9 ± 9.5	20.7 ± 9.6	21.2 ± 9.2	0.5
Quality of life	69.6 ± 21.4	69.5 ± 21.9	69.5 ± 20.7	0.9
Pain severity	6.1 ± 2	6.1 ± 2.1	6.1 ± 1.9	0.7
Pain acceptance	39.1 ± 19.07	38.8 ± 18.5	39.6 ± 19.8	0.6
pain catastrophizing	27.3 ± 12	27.4 ± 11.8	27.1 ± 12.2	0.7
Sleep disturbance	11.1 ± 4.6	11.3 ± 4.05	11.2 ± 4.5	0.5

### Correlational matrix among variables

Pearson’s correlation (univariate correlations) between QoL, pain severity, anxiety, depression, pain acceptance, pain catastrophizing, and sleep disturbance are presented in Table 3.

**Table 3:** Correlation matrix among variables

Variables	QoL	Pain severity	Anxiety	Depression	Pain acceptance	Pain catastrophizing
QoL	1					
Pain severity	− 0.321**	1				
Anxiety	− 0.352**	0.135**	1			
Depression	− 0.484**	0.154**	0.273**	1		
Pain acceptance	0.409**	− 0.104*	− 0.3**	− 0.289**	1	
Pain catastrophizing	− 0.426**	0.271**	0.252**	0.273**	− 0.295**	1
Sleep disturbance	− 0.367**	0.154**	0.338**	0.275**	− 0.356**	0.271**

^**^Significant at level P < 0.01, *Significant at level P < 0.05

According to the Table 3, QoL increases with increasing pain acceptance. There is also an inverse relationship between anxiety, depression, pain catastrophizing, pain severity, sleep disturbance and QoL. Interactions between all of the variables with each other are significant, except for the correlation between pain severity and pain acceptance. Depression has the strongest negative correlation (r =  − 0.484) with QoL. Also, Pain severity has the weakest negative correlation (r =  − 0.32) with QoL.

Normality and homoscedasticity of the error distribution were examined before operating the regression. The Kolmogorov–Smirnov analysis showed that the variables had a normal distribution. Homoscedasticity. In the current data, the residuals and the variance of the residuals were the same for all predicted variables. None of the distances were bigger than or equal to Chi‑square, so there were no multiple outliers among the data.

### Regression analysis

A step‑by‑step multiple regression analysis was conducted to predict life quality (criterion variable) based on pain severity, pain catastrophizing, pain acceptance, depression, anxiety, and sleep disturbance (predictive variables). Six models were implemented in which the sixth one demonstrated the highest R square.

According to Table 4, a significant regression equation was found: (F (10,1110) = 74.1, P < 0.001) with an R2 = 0.42, which confirmed that the model adequately fits the data. Overall, the results showed that all independent variables significantly predicted quality of life (P < 0.05).

**Table 4:** Analysis of variance of model (Anova ^a^)

	Sum of squares	df	Mean square	F	Sig
Regression	218,691.775	11	19,881.070	74.108	.000
Residual	297,245.936	1108	268.273		
Total	515,937.711	1119			

^a^Dependent Variable: quality of life

^b^Predictors: (Constant), Insulin treatment, pain catastrophizing, age, education, sex, diabetes duration, sleep, pain acceptance, anxiety, pain severity and depression

Table 5 assesses the regression analysis of variables regarding QOL. Based on the R square measure, the current model explained approximately 42% of the variance. This model takes the form of a statistical equation.

$$\begin{aligned} {{\text{Ypred}} = {\text{a}} + {\text{b}}1*1 + {\text{b}}2*2 + {\text{b}}3*3 + {\text{b}}4*4 + {\text{b}}5*5 + {\text{b}}6*6} \hfill \\ {\text{Ypred}} = \, 110.8 \, - \, 0.71*{\text{Depression}} - 0.353*{\text{Pain catastrophizing}} \\ & + \, 0.207*{\text{Pain acceptance}}{-}1.67*{\text{Pain Severity}}{-}0.549*{\text{sleep disturbance }} \\ {-}0.219 \, *{\text{Anxiety}}. \\ \end{aligned}$$

**Table 5:** Coefficients ^a^ of regression model

Model	Unstandardized coefficients		Standardized coefficients	t		Sig
	B	Std. error	Beta			
(Constant)	110.878	4.769	N/A	23.250		.000
Pain severity	− 1.671	.257	− .157	− 6.509		.000
Pain catastrophizing	− .353	.045	− .197	− 7.773		.000
Pain acceptance	.207	.029	.184	7.143		.000
Depression	− .710	.064	− .283	− 11.016		.000
Anxiety	− .219	.058	− .097	− 3.788		.000
Sleep disturbance	− .549	.120	− .118	− 4.555		.000

^a^Dependent Variable: quality of life

### Psychological symptoms prevalence

With a cut-off point of 14, the prevalence of depressive symptoms was 63.2%. Scores are distributed in the Spectrum of Depression, which are reported in Table 6.

**Table 6:** Spectrum of depression in the study population

Depression category	Range	No. of patients	Percentage (%)
Non depression	0–13	412	36.8
Mild depression	14–19	186	16.6
Moderate depression	10–28	328	29.3
Severe depression	29–63	194	17.3

As stated in the method section, the cut-off point for clinically significant anxiety on the BAI is 16. The prevalence of anxiety in patients with PDN is 62.1%. Scores are distributed in the Spectrum of anxiety, which are reported in Table 7.

**Table 7:** Spectrum of anxiety in the study population

Anxiety category	Range	No. of Patients	Percentage (%)
Nothing anxiety	0–16	204	18.2
Mild anxiety	17–35	327	32.8
Moderate and severe anxiety	36–63	392	35.4

Also, regarding the comorbidity between depression and anxiety, 47% of patients with PDN have the comorbidity symptoms of depression and anxiety. Regarding sleep problems, the cut-off point for sleep problems is 5. Therefore, according to data, the prevalence of sleep problems is 85.5%. That is, 85% of patients with PDN have significant problems related to sleep needing to follow.

## Discussion

The present study has predicted the quality of life in patients with PDN based on pain severity, pain catastrophizing, pain acceptance, Depression, Anxiety, and sleep disturbance. Also, in this research, we estimated the prevalence of anxiety, depression, and sleep disorders/symptoms in Iranian patients with PDN. Correlation analyses demonstrated that QoL has a significant correlation with all predictive variables. The results collected from the regression analyses also showed that these variables could predict nearly half of the QoL PDN patients' variance. Depression has the most contribution to this equation. Pain catastrophizing achieved the second position, and the latest position belongs to anxiety.

This is the first study for assessing the most prevalent psychological variables as predictors of QoL in PDN. However, the results are in line with previous researches. For example, a study examined correlations between anxiety, disability, and QoL in patients with PDN. Results showed that anxiety can reduce QoL both directly and indirectly (with increasing pain-related disability) in PDN [10]. In another study, pain-related factors reduced QoL in patients with diabetes [34]. Furthermore, Cherif et al. (2020) found that depression symptoms extensively reduced QoL in patients with PDN. Moreover, they found that depression was positively correlated with pain severity [19]. Also, Pain Catastrophizing Is Independently Associated with QoL in medical contexts [35].

Our research showed that pain catastrophizing has more portion than pain severity in QoL. Previous results are in line with this result too. For example, Lame et al. (2005) found that QoL is more correlated with beliefs about pain (especially pain catastrophizing) than pain severity in chronic pain [36]. These researches have similar results with the current paper. Recently, the first and corresponding author of this paper conducted extensive research in PDN. In our published works for that project, we found that increasing sleep quality and pain acceptance and reducing pain catastrophizing and depression can reduce PDN symptoms also increase response to treatment and consequently improve QoL in patients with PDN [9, 12].

Based on the present study, depression is the strongest variable in predicting QoL. Depression largely leads to physical disability. This disability reduces social participation, medical/psychological rehabilitation, and enjoyable activities. These factors, in turn, increase depression. Thus, the patient engages in a vicious cycle of depression and disability, which leads to severe depression and an extensive reduction in QoL. It has also been shown that the symptoms of depression lead to increased anxiety (anxiety symptoms) and sleep disturbance [37].Thus, depression also reduces the quality of life by changing the quality of sleep and increasing anxiety and therefore has a major role in reducing the quality of life [38].After the depression, variables related to pain perception (pain acceptance and pain catastrophizing) play an important role (even more than pain intensity) in reducing the quality of life. Based on the results of previous studies, this level of estimated disability due to pain is much higher than the actual level [10].Catastrophizing pain increases anxiety by increasing estimation of the level of disability, leading to making no effort to improve the quality of life. Catastrophizing pain also reduces self-efficacy, and the person thinks he is very disabled, so even healthier aspects, despite severe pain cause a wide reduction in quality of life [14, 35, 36]. Also, in patients with PDN, the prevalence of sleep disturbances, depression, and anxiety are 85.5%, 68.2%, and 62.1%, respectively. Also, comorbid depression and anxiety were found in 47% of patients. These results are in line with previous studies in other countries. For example, in Tunisian patients with PDN, depression, and anxiety rates are 65.6% and 73.7% [19]. Also, in the USA, the anxiety rate in PDN is 57% which is close to our results [39]. Also, various researches in chronic pain found that sleep disturbances rate range between 50 and 80% [40, 41]. So, in Iranian patients with PDN, sleep disturbances are higher than in almost all countries. Therefore an especial focus in the course of treatment plans is needed.

## Limitation and future directions

The current study has several limitations. Our research is cross-sectional, and the results cannot be generalized to long-term patients’ life. Also, based on financial limitation we could not assess MRI evaluation. Future researches can assess the effectiveness of biopsychosocial treatment based on our equation. Also, researchers can assess the prevalence of other psychiatric problems in PDN and classify them by age. Another research topic can compare this equation before and after successful treatment and analyze the network shape of symptoms to determine core symptoms. Finally, future researches can assess network analysis about different psychiatric problem in patients with PDN.

## Conclusion

The current paper found that depression, pain catastrophizing, pain acceptance, pain severity, sleep disturbance, and anxiety determine 42% of QoL in patients with PDN. The order to determine a role in QoL from the strongest to the weakest is depression, pain catastrophizing, pain acceptance, pain severity, sleep disturbance, and anxiety. Also, in patients with PDN, the prevalence of sleep disturbances, depression, and anxiety are 85.5%, 68.2%, and 62.1%, respectively. Also, comorbid depression and anxiety were found to be 47%. So, it is essential to focus on these psychiatric symptoms in PDN to increase QoL and treatment outcomes.

## Data Availability

Data of participants who consented to the public sharing of data are accessible from the corresponding author upon reasonable demands.

## Abbreviations

QoL: Quality of life
PDN: Painful diabetic neuropathy
NeuroQoL: Quality of life questionnaire
PCS: Pain catastrophizing scale
CPAQ: Chronic pain acceptance questionnaire
PSQI: Pittsburgh sleep quality index
