# Correction to: Predicting the quality of life based on pain dimensions and psychiatric symptoms in patients with Painful diabetic neuropathy: a cross-sectional prevalence study in Iranian patients

**DOI:** 10.1186/s12955-022-01939-5

**Published:** 2022-02-24

**Authors:** Mohammadreza Davoudi, Parnian Rezaei, Fereshteh Rajaeiramsheh, Seyed Majid Ahmadi, Amir Abbas Taheri

**Affiliations:** 1 Department of Clinical Psychology, Faculty of Behavioral Science, University of Social Welfare and Rehabilitation Sciences, Tehran, Iran; 2 Department of Clinical Psychology, Shahid Beheshti University, Tehran, Iran; 3 Department of Internal Medicine, School of Medical, Yasouj University of Medical Sciences (YUMS), Yasouj, Iran; 4 Department of Clinical Psychology, Faculty of Medicine, Kermanshah University of Medical Sciences, Kermanshah, Iran

## Correction to: Health Qual Life Outcomes (2021) 19:49 https://doi.org/10.1186/s12955-021-01697-w

The original article contains some errors which we wish to correct.

Firstly, there is an error in the affiliation given for Amir Abbas Taheri. The correct affiliation should read: “Department of Clinical Psychology, Faculty of Medicine, Kermanshah University of Medical Sciences, Kermanshah, Iran”.

Secondly the reference for ethics committee approval is incorrect as shown. The updated “Ethics approval and consent to participate” section should read as follows: “Written informed consent (about participation in the study) was received from all patients before the beginning of the study. The scales used in this research were all filled anonymously and a numeric code was used. This project was assessed and certified by the ethics committee of Kermanshah University of medical science (IR.KUMS.REC.1397.939)”.

Finally, in the "Results" section, the first sentence of the second paragraph should read: “*All of the patients were residents of Tehran, Iran*”.

These corrections do not affect the results and conclusions of the study. The authors would like to apologize for any inconvenience to readers.

## References

[1] Davoudi M (2021). Predicting the quality of life based on pain dimensions and psychiatric symptoms in patients with Painful diabetic neuropathy: a cross-sectional prevalence study in Iranian patients. Health Qual Life Outcomes.

